# Editorial: Morphological and functional imaging: a combined approach

**DOI:** 10.3389/fmed.2023.1283284

**Published:** 2023-09-14

**Authors:** Domenico Albano, Giulia Besutti

**Affiliations:** ^1^Nuclear Medicine Department, ASST Spedali Civili of Brescia, Brescia, Italy; ^2^Università degli Studi di Brescia, Brescia, Italy; ^3^Radiology Unit, Azienda USL-IRCCS di Reggio Emilia, Reggio Emilia, Italy; ^4^Department of Medical and Surgical Sciences, University of Modena and Reggio Emilia, Modena, Italy

**Keywords:** imaging, PET, CT, MRI, hybrid imaging, nuclear medicine

Hybrid imaging, namely, the fusion of different imaging techniques in a single technology, and combined imaging, e.g., the merging of information derived from different imaging techniques, are increasingly used for disease diagnosis, characterization, and prognosis prediction. In fact, it is clear that hybrid and combined imaging give the best chance to study several oncological and not oncological diseases due to the possibility of having different information: functional and metabolic data typically from positron emission tomography/computed tomography (PET/CT) studies (for example, glucose metabolism studied with [18F]FDG, or phospholipidic metabolism studied with [18F]choline) and morphological data derived by computed tomography (CT) and/or magnetic resonance imaging (MRI). MRI itself, by combining different sequences, may also provide functional data besides purely morphological information. This Research Topic included six articles related to applications of morphological and functional imaging techniques in different fields of medicine. This topic consisted of four original articles, one review, and one case report.

In a prospective study, Darcot et al. compared CT and several ultra-short eco time MRI sequences (ultra-short eco time under high-frequency non-invasive ventilation, UTE-HF-NIV, and in free-breathing, UTE-FB, and volumetric interpolated breath-hold examination at full inspiration, and VIBE-BH) for the detection and volumetric assessment of lung nodules. The diagnostic accuracy of UTE-HF-NIV and UT-FB compared with CT is sub-optimal, with a detection rate of 35% for nodules ≥4 mm and 50% for nodules ≥6 mm. When comparing volumetric measurements, an excellent agreement was observed between CT and UTE-HF-NIV, with a small overestimation of 13.2% by UTE-HF-NIV. Less concordant findings were derived comparing CT and VIBE-BH with an overestimation of 28.8% by VIBE-BH.

Another study included in this topic was based on the role of MRI in type-2 diabetic (T2D) patients. Bouazizi et al. included 36 T2D patients and 29 healthy controls who underwent cardiac and aortic MRI exams at 1.5 tesla. Applying a specific software, epicardial adipose tissue (EAT), ascending aorta (AA) and descending aorta (DA) areas, aortic flow volumes (AA and DA global; forward and backward flow volumes), and distensibility were measured by MRI. They demonstrated that EAT was significantly higher in T2D patients than in controls and was negatively correlated to AA distensibility and positively to the backward flow volume. In the multivariate analysis, the presence of T2D and AA backward flow to forward flow volume ratio are independently associated with EAT.

Beyond CT and MRI, nuclear medicine imaging tools may also be helpful in different settings. In a bicentric study, Jacquet-Francilon et al. investigated the usefulness of [18F]choline PET/CT in detecting hyperfunctioning parathyroid glands in patients with hyperparathyroidism. Particularly, they focused on the semiquantitative parameters, like SUVmax, liver ratio, thyroid ratio, and size ratio. Among them, SUVmax and liver ratio were the best predictors of the final diagnosis (adenoma vs. hyperplasia) with a specific cut-off of 4.12 (AUC = 0.858) and 27.4 (AUC = 0.790), respectively. Moreover, they demonstrated that the detection rate of PET was significantly correlated with PTH level: the detection rate was 56, 75, and 87.5%, respectively, for PTSH <70 ng/mL, 70–120 ng/ml, and >120 ng/mL.

In another article, Ngoc Huynh et al. included 238 patients with head and neck squamous cell cancer from two European centers. They evaluated the prognostic role of conventional radiomics and deep learning radiomics derived by pre-treatment [18F]FDG PET and CT. The main results of this study were that deep learning radiomics using image-based convolutional neural network (CNN) models outperformed conventional radiomics and clinical models with regard to both performance and generalizability for the prediction of overall survival and disease-free survival. Combining these image-based models with clinical data and conventional radiomics features increased performance. Besides, image-based CNN models trained without tumor and node contours achieved as high or very similar performances as models trained with contours.

Overall, morphological and functional imaging techniques are now considered fundamental in the diagnosis and management of patients affected by COVID-19. An expert review included in this Research Topic written by Varadarajan et al. describes deeply the role of the main imaging tools (radiogram, ultrasound, CT, MRI, and [18F]FDG PET). Beyond a detailed description of the main findings of each technique, the authors describe the main “imaging” evidence for each district (respiratory tract, cardiovascular system, neurological system, and gastrointestinal system). With this narrative review, it emerges clearly that imaging tools in combination with clinical examination and biomarkers may give an exhaustive assessment and management of organ dysfunction and damage in patients affected by COVID-19.

Finally, Huang et al. analyzed the role of multimodal imaging (MRI and [18F]FDG PET/CT) in two young men with nasal sinus alveolar rhabdomyosarcoma. This is a disease with a poor prognosis due to the difficulties of reaching a precise and early diagnosis. MRI and PET/CT may give crucial information in the assessment of the extent of the disease and the presence of metastases, in the choice of the best treatment (guiding surgery) and help during the follow-up.

The first message we can derive from this Research Topic is that the potential applications of combined imaging are extremely wide, involving systemic as well as organ-specific diseases and both oncological and non-oncological diseases. Also, the purposes of the application of multimodality imaging largely vary from disease detection and diagnosis, measurement and characterization, and patient outcome prediction. In conclusion, the articles included in this Research Topic clearly highlight the bright future of hybrid and combined imaging in different settings.

## Author contributions

DA: Writing—original draft, Writing—review and editing. GB: Writing—original draft, Writing—review and editing.

